# The insufficiency of recreational exercises in improving cardiovascular fitness: an investigation of ventricular systolic and diastolic parameters and left atrial mechanical functions

**DOI:** 10.1186/s12872-023-03508-0

**Published:** 2023-10-04

**Authors:** Alkame Akgümüş, Ahmet Kurtoğlu, Engin Aydın, Ahmet Balun, Bekir Çar, Özgür Eken, Monira I. Aldhahi

**Affiliations:** 1 Department of Cardiology, Medical Faculty, Bandirma Onyedi Eylul University, Bandırma, 10200 Turkey; 2 Department of Coaching Education, Faculty of Sport Science, Bandirma Onyedi Eylul University, Bandırma, 10200 Turkey; 3 Department of Pediadrics, Medical Faculty, Bandirma Onyedi Eylul University, Bandırma, 10200 Turkey; 4 Department of Physical Education and Sport Teaching, Faculty of Sport Sciences, Bandirma Onyedi Eylul University, Bandırma, 10200 Turkey; 5 Department of Physical Education and Sport Teaching, Faculty of Sports Sciences, Inonu University, Malatya, Turkey; 6 Department of Rehabilitation Sciences, College of Health and Rehabilitation Sciences, Princess Nourah bint Abdulrahman University, P.O. Box 84428, Riyadh, 11671 Saudi Arabia

**Keywords:** Athletics, Recreation, Sports, Electrocardiography, Atrial mechanical functions, Ventricular function, Diastolic, Systolic, Resistance exercise

## Abstract

**Aim:**

This study aimed to compare the left ventricular (LV) systolic and diastolic parameters and left atrial (LA) mechanical functions of individuals engaging in recreational sports and resistance exercises on a weekly basis.

**Methods:**

A total of 43 male amateur athletes were included in this study, of which 24 performed resistance exercises (REs) (29.70 ± 8.74 year, weight: 81.70 ± 12.64 kg, height: 176.05 ± 7.73 cm, BMI: 27.64 ± 4.97 kg/m^2^), and 19 participated in recreational football training and were included in the recreational sports group (31.73 ± 6.82 year, weight: 86.00 ± 18.52 kg, height: 178.62 ± 4.95 cm, BMI: 25.55 ± 3.42 kg/m^2^). The exercises were standardized according to the weekly exercise frequency and volume. After recording the participants’ demographic information, the LV systolic and diastolic parameters and LA mechanical functions were measured using echocardiography (ECHO) and Tissue Doppler Imaging.

**Results:**

Significant differences were observed in various cardiac parameters between the recreational sports group (REG) and resistance exercise Group (RSG). Specifically, the left ventricular (LV) diastolic diameter, LV end diastolic volume index (LVEDVi), and stroke volume index were notably higher in the REG compared to the RSG (t = 2.804, p = .010, effect size (ES) = 2.10; t = 3.174, p = .003, ES = 0.98; t = 3.36, p = .002, ES = 1.02, respectively). Notably, the RSG exhibited higher values for LV mass index (LVMi) and isovolumic relaxation time (IVRT) than the REG (t = 2.843, p = .007, ES = 0.87; t = 2.517, p = .016, ES = 0.76) in terms of LV systolic and diastolic parameters. Regarding left atrial (LA) mechanics, the REG demonstrated increased LA total emptying volume index, LA maximum volume index, LA volume before systole measured at the onset of the p-wave index, and conduit volume index compared to RSG (t = 2.419, p = .020, ES = 0.75; t = 2.669, p = .011, ES = 0.81; t = 2.111, p = .041, ES = 0.64; t = 2.757, p = .009, ES = 0.84, respectively).

**Conclusion:**

Our study revealed significant variations in LV and LA functions between REG and RSG. Our data suggest that REs led to substantial cardiac remodeling, altering myocardial structure and function. In contrast, the effect of recreational exercise on cardiac adaptation was less pronounced than that of resistance exercise. Consequently, we propose that individuals engaging in recreational exercise should consider modalities that impose higher cardiovascular demand for more effective cardiac conditioning.

## Introduction

Physical fitness and cardiovascular health are of paramount importance for athletes seeking to optimize their performance and overall well-being [1]. Cardiovascular fitness plays a critical role in an athlete’s ability to exert maximal effort. While structured training programs and rigorous workouts are essential components of an athlete’s routine, the role of recreational exercises in improving cardiovascular fitness has gained considerable attention. Recreational activities play a vital role in promoting physical fitness and overall well-being among individuals, including amateur athletes [2]. Recreational sports involve physical movements that occur during leisure time and trigger physiological bodily systems. These exercises include activities that are performed regularly, whether in adulthood or youth, and at the amateur or professional level [3]. As part of a larger spectrum of physical activity choices, individuals participate in recreational activities to promote their emotional and mental well-being by emphasizing enjoyment and relaxation, in contrast to exercises that focus on structured physical activity [3]. Engaging in regular recreational activities has been associated with numerous cardiovascular benefits, including enhanced exercise capacity and a reduced risk of cardiovascular diseases [4, 5]. Although the benefits of recreational exercise on cardiovascular fitness have been widely acknowledged [6–8], their specific effects on ventricular systolic and diastolic parameters and left atrial (LA) mechanical functions in amateur athletes have not been thoroughly investigated.

Over the last decade, growing evidence has shown that recreational football is a multifaceted exercise that offers a range of advantages beyond structured training sessions. Milanović et al. [9] meta-analyzed 17 studies and reported that recreational football produces large improvements in maximal oxygen consumption compared to strength training and no-exercise controls. Another meta-analysis demonstrated multiple broad-spectrum benefits of recreational football on health-related physical fitness compared with non-exercise controls, including improvements in blood pressure, resting heart rate, fat mass, low-density lipoprotein cholesterol, and countermovement jump performance [7]. Such activities provide an opportunity for athletes to engage in physical activities that are enjoyable and less regimented, thereby promoting mental well-being and reducing stress levels [1, 8]. Moreover, recreational football often involves dynamic movement patterns, varying intensities, and different energy systems, leading to a diverse set of physiological stimuli. This diversity in exercise patterns can contribute to improved central hemodynamics, as it challenges the cardiovascular system in different ways, thereby promoting adaptability and efficiency. However, the specific effects on the ventricular and atrial function parameters require further investigation.

It has been reported that recreational exercises may influence ventricular systolic and diastolic parameters, as well as LA mechanical functions [10, 11]. Physical activity has been found to result in an increase in LV end-diastolic volume (LVEDV). Additionally, it tends to augment the total peripheral vascular resistance (TPR) as well as blood flow towards the extremities [12]. Yet, little is known about the type of exercise performed. Traditional high-load (HL) resistance exercise (RE) routines, comprising 8–12 repetitions, at approximately 70–80% of one repetition maximum (1RM), have been shown to generate adaptations that lead to muscle improvement [13]. Both high-load (HL) and moderate-load (ML) exercise protocols comprising of 25–35 repetitions (approximately 30–50% of IRM) performed until volitional fatigue were found to stimulate hypertrophic adaptations [14]. Thus, regular exercise has been associated with numerous cardiovascular benefits, including improved cardiac function, increased cardiac output, and enhanced exercise capacity [15]. Although aerobic exercise is widely recognized for its cardiovascular benefits [15], there is growing interest in investigating the comparative effectiveness of engagement in regular recreational football and resistance exercises (REs) on cardiac adaptation. Recreational football combines the elements of both aerobic and anaerobic exercises, making it an appealing exercise modality to explore along with RE. Comparing the effects of RE and recreational football on cardiac adaptation allows for a comprehensive understanding of the potential cardiovascular adaptations associated with these exercise modalities. Therefore, this study aimed to investigate the potential effect of recreational football training compared to resistance exercises on cardiac adaptation among amateur athletes. Specifically, we examined the effect of recreational exercises on ventricular systolic and diastolic parameters, as well as LA mechanical function using echocardiography (ECHO) and Tissue Doppler Imaging (TDI). The ECHO and TDI are valuable tools for assessing cardiac adaptations because they allow the evaluation of structural and functional changes in the heart [16]. It is a well-known method for imaging changes in shape, thickness, ventricular and aortic diameters, velocity, and many diagnostic factors in clinical diseases [16]. Understanding the effects of exercise on ECHO parameters is important for optimizing exercise prescriptions, monitoring cardiovascular health, and guiding clinical decision making. Additionally, understanding these distinct effects can provide valuable insights for designing exercise programs tailored to the specific goals and needs of amateur athletes. Moreover, it can contribute to the existing body of knowledge on the benefits of recreational exercise and resistance training on cardiac function, potentially informing training recommendations for individuals seeking to improve their cardiovascular fitness and overall well-being.

## Methods

### Study design and participants

In this cross-sectional study, the required sample size was estimated based on a previous study conducted by Kurtoğlu et al. [17] in which the ECHO findings of football players was reported to be 50.91 ± 4.01 mm. Thus, we performed a power analysis using a significance level (α) of 0.05, power (1-β) of 0.80, and effect size of 0.86. The analysis indicated that a minimum of 18 participants per group would be needed in our study.

**Fig. 1:**
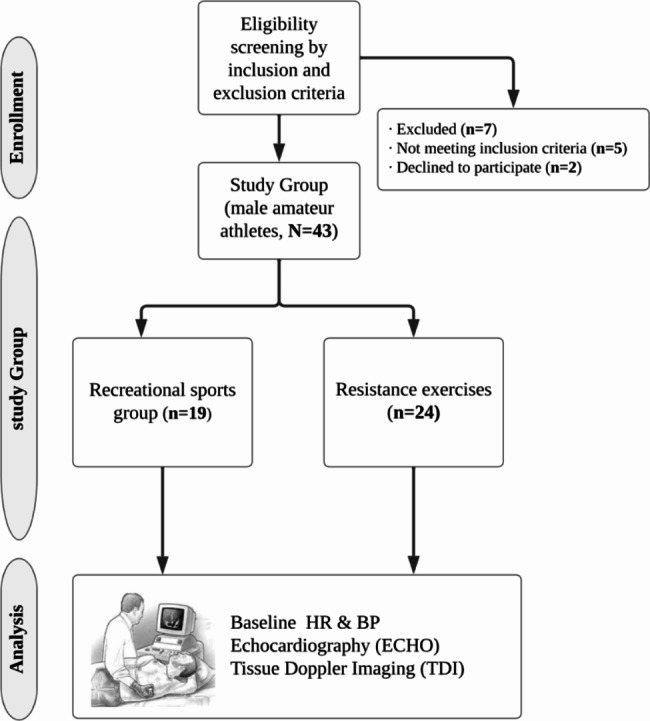
Study flow diagram

A nonprobability convenience sampling method was used in this study. The inclusion criteria were male individuals without pre-existing health conditions who engaged in resistance training and recreational football at least twice weekly for a duration of one year. Participants who engaged in resistance training that involved the progressive use of various resistive loads, movement velocities, and training modalities were included in the resistance exercise group (REG). The training modalities include weight machines, free weights (e.g., barbells and dumbbells), elastic bands, medicine balls, or plyometrics performed twice a week. Participants who engaged in recreational football training for at least twice a week were included in the recreational sports group (RSG). We excluded male participants with hypertension, tachycardia or bradycardia, developmental disorders (e.g., thyroid and heart valve disorders), and those who had already undergone surgery for a heart problem. We also excluded participants who reported any history of sleep disorders, who did not comply with the researcher’s instructions, or who were found to have health problems during testing. In this context, a total of 24 male participants who performed REs (REG) for at least 2 days a week, and 19 male participants who played football for recreational purposes (RSG) at least 2 days a week were included in our research. The study participants self-reported an average duration of sports experience of 4 ± 2.5 years. All participants were assessed after 8 h of sleep and were instructed not to eat or drink other than water for at least 3 h before taking part in the testing. The flow diagram of the study is illustrated in Fig. 1.

This study was conducted in accordance with the principles outlined in the Declaration of Helsinki and was approved by both Bandirma Onyedi Eylul University (BU) and the Human Research Ethics Committee of BU (approval number: 2022/222). Written informed consent was obtained after participants were informed of the purpose and details of the study.

### Study procedure and measurement

#### Body surface area

After determining the demographic characteristics of the participants, their height and weight were measured and used to calculate body mass index (BMI) using the following formula: body weight (kg)/height squared (m²). Body surface area (BSA) was calculated according to the following formula: body weight (kg) 0.425 × height (cm) 0.725 × 0.007184 [18].

#### Blood pressure

Blood pressure was measured by a cardiologist after 9 min of complete passive rest following the standardized method of measurement [19]. Systolic and diastolic blood pressures were measured using a stethoscope and a sphygmomanometer (Erka Perfect Aneroid / Germany).

#### Echocardiographic measurements (ECHO)

The ECHO evaluations were performed by a specialist cardiologist in the cardiology clinic. All tests were performed at the same time of the day (morning). Participants were asked to take eight hours of sleep before the measurements. All ECHO examinations were performed using the Vivid T8 device and 3ScRS transducer (GE Medical System, Milwaukee). All measurements were performed according to the recommendations of the American Society of Echocardiography Guidelines [20]. Echocardiographic images and recordings were obtained in the parasternal long-axis, apical four-chamber, and apical two-chamber views in the left decubitus position and at rest [21]. The following 2-dimensional and M-mode echocardiographic parameters were measured: aortic diameters in systole (ADs) and diastole (ADd), left ventricular end-diastolic diameter (LVDd in mm), left ventricular end-systolic diameter (LVDs), interventricular septal thickness (IVST), and posterior wall thickness (PWT).

The left ventricular end-diastolic volume (LVEDV), left ventricular end-systolic volume (LVESV), stroke volume (SV), and ejection fraction (EF) were measured in the apical four-chamber view by the modified Simpson method [22]. Pulsed wave (PW), early diastolic flow velocity (E), late diastolic flow parameter (A), E/A ratio, ejection time (ET), isovolumic relaxation time (IVRT), isovolumic contraction time (IVCT), and transmissible flow parameters were measured. Tissue Doppler imaging of annulus motion was measured from lateral mitral annulus and peak early systolic (Sm), peak early diastolic (Em), and peak late diastolic (Am) velocities.

#### Tissue doppler imaging (TDI)

The LV lateral wall in apical 4-chamber views from the mitral lateral annulus was used for TDI. A clear image signal was obtained by adjusting the TDI filter and Nyquist cutoff to a value of 16–20 cm/s. The left ventricular mass index (LVM-I) was calculated using the Devereux formula [23]. The aortic systolic and diastolic diameters were obtained from the recordings made after the insertion of the M-mode rod through the area of the ascending aorta 3 cm distal to the aortic valve. The systolic and diastolic diameters were measured in the area corresponding to the R peak of the ECG from the point of maximum forward motion in the aortic curve. The measurements were repeated for three heartbeats and the average value was determined [24]. The aortic strain (AS) and aortic distensibility (AD) were used as aortic elasticity parameters. The following formula was used to calculate these parameters:

Aortic Strain (%) = [(systolic diameter-diastolic diameter) x 100] /diastolic diameter.

Distensibility (10 − 6.cm-2.dyn-1) = 2 (aortic strain ) / (systolic pressure-diastolic pressure) [25].

Left atrial volumes were calculated from apical four-chamber and two-chamber views using the biplane field length method. The maximum left atrial volume (LAVmax) was measured when the mitral valve was fully open, minimum left atrial volume (LAVmin) was measured when the mitral valve was fully closed and left atrial volume before systole was measured at the onset of the p wave (LAVp) on the electrocardiogram. All measurements were repeated during three consecutive heartbeats and averaged. The LAVmax index was determined by dividing the maximum volume of the left atrium (LAVmax) by body surface area. All volumes were corrected by dividing by the LAVmax index. Left atrial function was determined using the following formula [26].

- LA passive emptying volume (LAPEV) = LAV max – LAVp.
- LA passive emptying fraction (LAPEF) = LAPEV / LAVmax.
- LA active emptying volume (LAAEV) = LAVp – LAVmin.
- LA active emptying fraction (LAAEF) = LAAEV / LAVp.
- LA total emptying volume (LATEV) = LAVmax - LAVmin.
- LA total emptying fraction (LATEF) = LATEV / LAVmax.
- Conduction volume (CV) = Left ventricular stroke volume - (LAVmax-LAVmin).

All ECHO measurements were performed by two independent experts. The similarity between the data in both analyzes was determined as 96.8%.

### Statistical analysis

Statistical analyses were performed using SPSS 25.0 (SPSS, Inc., Chicago, IL, USA). Normality analysis of the data was performed using the Shapiro-Wilk test. Levene’s test was performed to determine the homogeneity of variances. The data were found to be normally distributed. For comparison of the mean differences between the groups in the outcomes of interest, the independent sample t-test was used. GraphPad Prism 8 was used to visualize the data. Cohen’s d effect size (ES) with 95% confidence interval was calculated to define the magnitude of pairwise comparisons for pre- and post-test. The ES magnitude was defined as follows: <0.2 = trivial, 0.2 to 0.6 = small effect, > 0.6 to 1.2 = moderate effect, > 1.2 to 2.0 = large effect, and > 2.0 = very large [27]. Statistical significance was set at an alpha level of 0.05.

## Results

**Table 1:** Demographic Information of Participants

Parameters	REG (n = 24)	RSG (n = 19)	p-value
	**M ± SD**	**M ± SD**	
Age (year)	29.70 ± 8.74	31.73 ± 6.82	0.411
Weight (kg)	81.70 ± 12.64	86.00 ± 18.52	0.373
Height (cm)	178.62 ± 4.95	176.05 ± 7.73	0.218
BMI (kg/m^2^)	25.55 ± 3.42	27.64 ± 4.97	0.111
Sport experience (year)	3.66 ± 2.88	4.52 ± 2.11	0.284
Training frequency (day)	2.62 ± 0.49	2.42 ± 050	0.192
BSA (m^2^)	2.00 ± 0.16	2.03 ± 0.23	0.614
SBP (mmHg)	122.87 ± 10.23	123.26 ± 10.25	0.902
DBP (mmHg)	73.45 ± 11.23	76.57 ± 8.34	0.319

^Abbreviations: BMI: Body mass index, TF: Training frequency (in a week), BSA: Body surface area, SBP: Systolic blood pressure, DBP: Diastolic blood pressure, REG: Resistance exercise group, RSG: Recreational sports group^

Table 1 shows the demographic characteristics, sports experiences and weekly exercise duration of the participants. The study findings revealed no significant variance among the groups in age, weight, height, sports experience, training frequency, BSA, BMI, Systolic blood pressure (SBP), and diastolic blood pressure (DBP) outcomes (p > .05). These results suggest that the groups share similar characteristics.

**Fig. 2:**
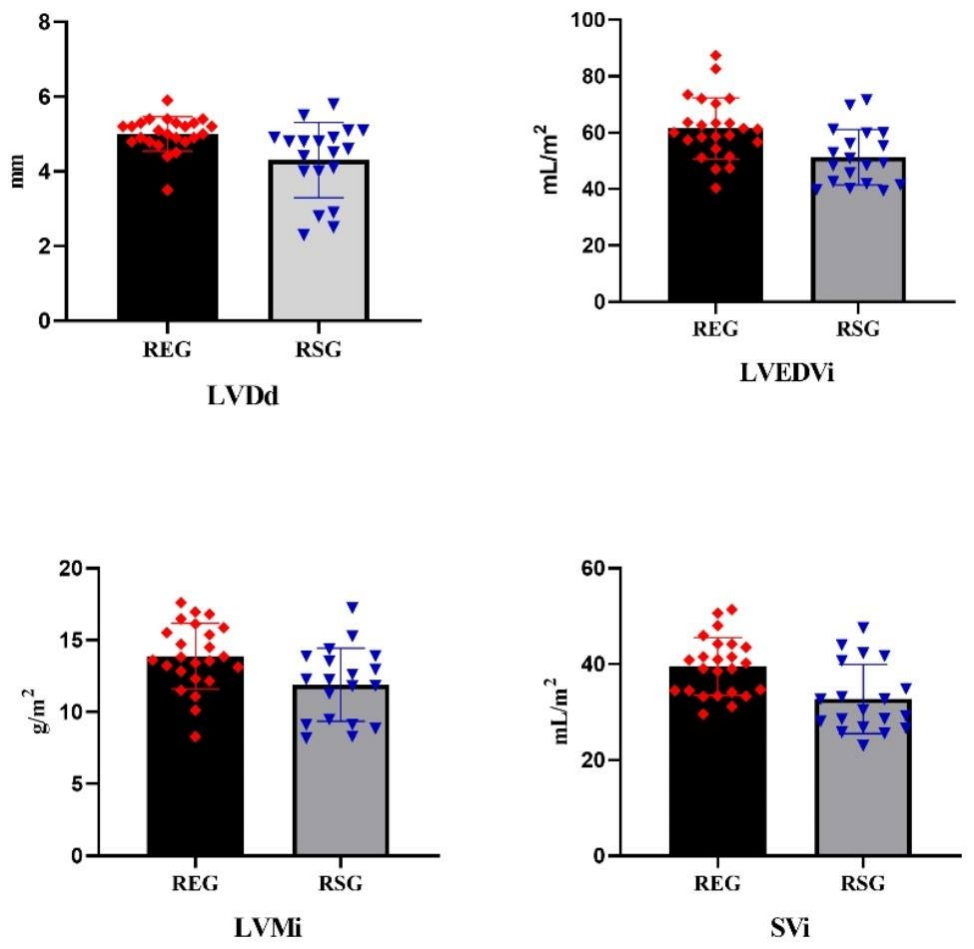
Description of the ECHO parameters across the groups

Table 2 shows the results of the routine ECHO parameters of the participants. It was observed that the values of the LV diastolic diameter (LVDd) (t = 2.804, p = .010, ES = 2.10), LVEDVi (t = 3.174, p = .003, ES = 0.98), and stroke volume index (SVi)(t = 3.36, p = .002, ES = 1.02) and LV mass index (LVMi) (t = 2.843, p = .007, ES = 0.87) were significantly higher in the REG than in the RSG (Fig. 2). However, there were no significant difference between the groups in ADd, ADs, LVDs, LVESVi, IVST, and PWT (p > .05). Figure 2. **Description of the ECHO parameters across the groups**.

**Table 2:** Examination of rutine ECHO parameters of participants

Parameters	REG (n = 24)	RSG (n = 19)	t	ES	p-value
	M ± SD	M ± SD			
ADd (mm)	22.58 ± 3.52	23.15 ± 2.03	-0.670	0.19	0.507
ADs (mm)	27.75 ± 3.59	27.68 ± 2.18	0.074	0.02	0.941
LVDd (mm)	50.04 ± 4.58	43.05 ± 1.00	2.804	2.10	0.010*
LVDs (mm)	32.83 ± 5.49	33.42 ± 5.56	-0.347	0.10	0.731
LVEDVi (mL/m^2^)	61.47 ± 10.93	51.25 ± 9.86	3.174	0.98	0.003*
LVESVi(mL/m^2^)	21.49 ± 6.82	18.93 ± 4.82	1.384	0.43	0.174
SVi (mL/m^2^)	39.49 ± 6.03	32.71 ± 7.18	3.361	1.02	0.002*
IVST (mm)	10.00 ± 1.53	9.63 ± 1.67	0.752	0.23	0.456
PWT (mm)	9.70 ± 1.69	9.31 ± 1.73	-0.195	0.06	0.846

^Abbreviations: ADd: aortic diameter diastole, ADs: aortic diameter systole, IVST: interventricular septal thickness, LVDd: left ventricular end−diastolic diameter, LVDs: left ventricular end−systolic diameter, LVEDVi: left ventricular end−diastolic volume index, LVESVi: left ventricular end−systolic volume index, PWT: posterior wall thickness, SVi: stroke volume index, REG: resistance exercise group, RSG: recreational sports group^.

^*Denotes statisitical significant (p<.05)^

**Table 3:** Left ventricular systolic and diastolic parameters of the participants

Variables	REG (n = 24)	RSG (n = 19)	t-value	ES	p-value
	M ± SD	M ± SD			
E (m/s)	0.81 ± 0.16	0.76 ± 0.13	0.944	0.34	0.351
A (m/s)	0.57 ± 0.13	0.58 ± 0.11	-0.350	0.08	0.728
ET (ms)	288.50 ± 23.99	303.47 ± 43.78	-1.429	0.42	0.161
IVCT (ms)	71.41 ± 12.98	73.52 ± 17.26	-0.458	0.13	0.650
IVRT (ms)	66.58 ± 7.27	60.48 ± 8.61	2.517	0.76	0.016*
Em (cm/s)	14.12 ± 2.67	14.52 ± 3.43	-0.431	0.13	0.669
Am (cm/s)	8.45 ± 2.10	8.52 ± 1.67	-0.115	0.03	0.909
Sm (cm/s)	9.58 ± 1.95	9.31 ± 2.35	0.407	0.12	0.686
LVEF (%)	65.87 ± 6.64	64.15 ± 7.59	0.790	0.24	0.434
E/A Ratio	1.48 ± 0.36	1.33 ± 0.24	1.431	0.49	0.160
E/Em Ratio	0.05 ± 0.13	0.05 ± 0.15	0.742	0.09	0.463

^Abbreviations: A: late diastolic flow parameter, Am: peak late diastolic, Em: peak early diastolic, ET: ejection time, E: early diastolic flow velocity, IVCT: isovolumic contraction time, IVRT: isovolumic relaxation time, LVEF: left ventricular ejection fraction, LVMi: left ventricular mass index, Sm: peak early systolic, REG: resistance exercise group, RSG: recreational sports group^

^*Denotes statistically significant (p<.05)^

LV systolic and diastolic parameters of the study participants are compared in Table 3. It was found that isovolumic relaxation time (IVRT) (t = 2.517, p = .016, ES = 0.76) was higher in the REG. However, no statistical significant differences were observed in the early and late diastolic flow parameter (E, A), ejection time (ET), isovolumic contraction time (IVCT), peak early diastolic (E), peak late diastolic (Am), peak early systolic (Sm), left ventricular ejection fraction (LVEF), E/A Ratio, and E/Em ratio (p > .05).

**Table 4:** Comparison of left atrial functions across the groups

Parameters	REG (n = 24)	RSG (n = 19)	t-value	ES	p-value
	M ± SD	M ± SD			
LAPEVi (mL/m^2^)	5.96 ± 2.29	4.69 ± 1.96	1.922	0.59	0.062
LAPEFi (mL/m^2^)	0.42 ± 0.11	0.43 ± 0.12	− 0.234	0.08	0.816
LAAEVi m(L/m^2^)	3.12 ± 1.12	2.55 ± 1.19	1.604	0.49	0.116
LAAEFi (mL/m^2^)	0.39 ± 0.10	0.37 ± 0.14	0.625	0.16	0.535
LATEVi (mL/m^2^)	9.00 ± 1.88	7.73 ± 1.46	2.419	0.75	0.020*
LATEFi (mL/m^2^)	0.64 ± 0.07	0.65 ± 0.07	-0.328	0.14	0.745
LAmini (mL/m^2^)	4.77 ± 1.28	4.16 ± 1.45	1.451	0.44	0.154
LAmaxi (mL/m^2^)	13.86 ± 2.28	11.90 ± 2.53	2.669	0.81	0.011*
LAPi (mL/m^2^)	7.90 ± 1.60	6.72 ± 2.05	2.111	0.64	0.041*
CVi (mL/m^2^)	30.40 ± 6.07	24.98 ± 6.77	2.757	0.84	0.009*

^Abbreviations: CVi: conduit volume index, LAAEF: left atrium active emptying fraction index, LAAEV: left atrium active emptying volume index, LAPEF: left atrium passive emptying fraction index, LAPEV−I: left atrium passive emptying volume index, LATEF−I: left atrium total emptying fraction index, LATEFSV−I: left atrium total emptying fraction index, REG: resistance exercise group, RSG: recreational sports group^

^*Denotes statistically significant results (p<.05)^

**Fig. 3:**
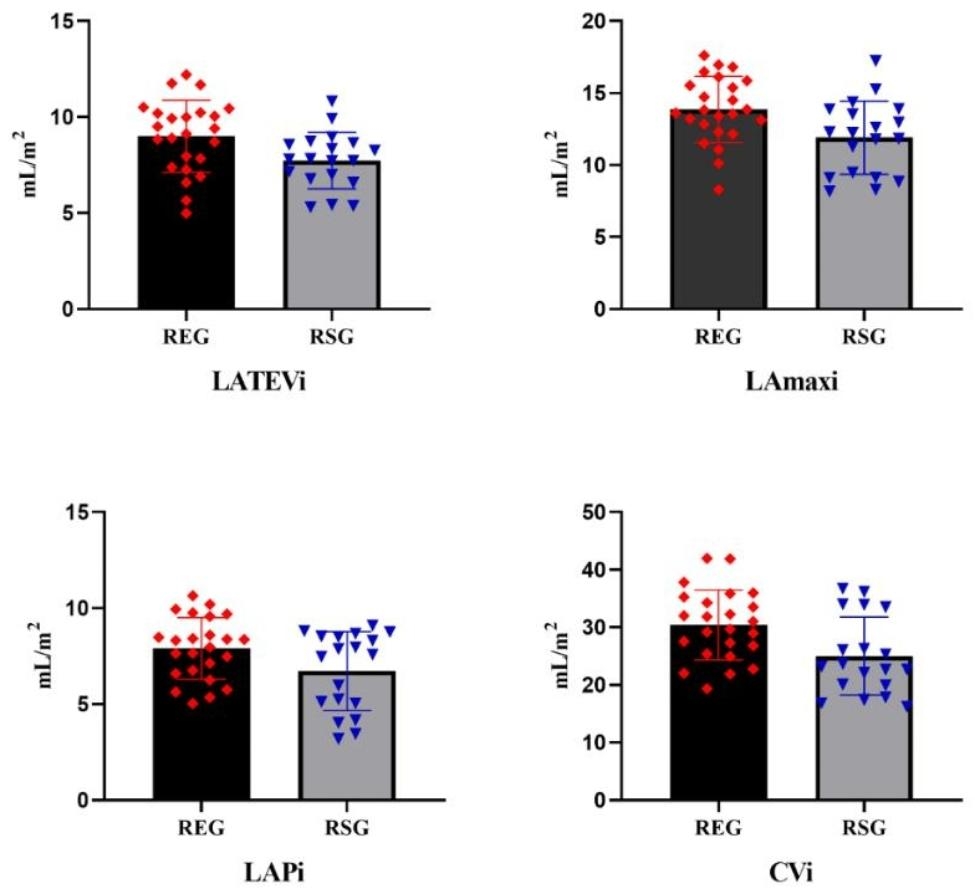
Comparison of the mean left atrial functions

The LA functions of the participants reported in Table 4. Figure 3 shows a higher mean value of the LA total emptying volume index (LATEVi), LA maximum volume index (LAmaxi), LA volume before systole measured at the onset of the p wave index (LAPi), and conduit volume index (CVi) in the REG than in the RSG (p < .05). However, there was no significant difference in left atrium passive emptying volume index (LAPEVi), LAPEFi, LAAEVi, LAAEFi, LATEFi.

Table 5 displays the results of aortic strain and distensibility among the participants. The statistical analysis reveals that there was no significant difference observed between the aortic strain and distensibility results among the participants (p > .05).

**Table 5:** Comparison of participants’ aortic strain and distensibility

Parameters	REG (n = 24)	RSG (n = 19)	t-value	ES	p-value
	M ± SD	M ± SD			
AS (%)	23.56 ± 8.22	19.83 ± 7.28	1.553	0.48	0.128
AD (cm^2^.dyn^− 1^.10^6^)	90.57 ± 13.58	87.01 ± 7.87	1.075	0.32	0.289

^Abbreviations: AD: aortic distensibility, AS: aortic strain, REG: resistance exercise group, RSG: recreational sports group^

## Discussion

In our investigation, we compared the LV systolic and diastolic parameters, as well as the left atrial (LA) mechanical functions, between individuals engaged in REs and those participating in recreational sports, with both groups maintaining similar weekly exercise durations. Primary outcomes illustrated that the resistance exercise group (REG) demonstrated elevated left ventricular end-diastolic diameter (LVDd), left ventricular end-diastolic volume index (LVEDVi), left ventricular mass index (LVMi), stroke volume index (SVi), and isovolumic relaxation time (IVRT) when compared to the recreational sports group (RSG). Furthermore, in regard to LA mechanical functions, late diastolic mitral annular velocity (LATEvi), maximum left atrial volume index (LAmaxi), left atrial pressure index (LAPi), and conduit volume index (CVi) were significantly augmented in the REG [33, 34]. To our knowledge, this study represents the first endeavor to elucidate the distinctions between left ventricular (LV) systolic and diastolic parameters and LA mechanical functions in individuals participating in both RE and recreational soccer.

Long-term and consistent exercise has been established to promote physiological adaptations within the cardiovascular system [28], [29], enabling acute responsiveness to increased demands imposed by skeletal muscles during physical activity [30]. As exercise that challenges the cardiovascular system becomes a habitual practice, adaptive alterations, such as cardiac hypertrophy and augmented contractility, are stimulated [30]. Current guidelines advocate for moderate exercise engagement, totaling a minimum of 150 min per week [31]. Nonetheless, the specific exercise modality that provides optimal health benefits to sedentary individuals remains a topic of ongoing debate. Our data highlight that REG participants exhibited superior LVDd, LVEDV, and SVi compared with their RSG counterparts, potentially as a consequence of elevated blood pressure, facilitating augmented skeletal muscle blood flow [32], diminished peripheral resistance, and enhanced cardiac contractile capacity [33]. Chronic exercise engagement prompts hemodynamic adaptations, normalizing LV wall tension via internal size adjustments and LV hypertrophy (LVH), while preserving or even elevating LV systolic and diastolic function [31] [34]. A previous study highlighted an increase in the LVEDd during heavy RE. It has been found that heavy RE significantly increases arterial blood pressure [35]. Keul et al. found that LVDd was higher in strength athletes than in long- and middle-distance runners [36]. Furthermore, the potential mechanism to increase end-diastolic volume is enhanced venous return to the heart and decreased resting heart rate, resulting in prolongation of diastole and increased diastolic filling. As a result, there is an increase in cardiac stroke volume [37]. Furthermore, it is known that long-term exercise increases the number of blood vessels in the muscles, resulting in a decrease in peripheral resistance and an increase in contractility [38].

It has been found that the LVMi was statistically elevated in the REG, which, considering equivalent LV wall thickness between the groups, is attributed to excessive LVEDV in the REG [39]. Corroborating the Morganroth hypothesis [40], our findings suggest that endurance training elicits eccentric LVH owing to volume loading and increased diastolic wall stress, whereas strength training predominantly induces concentric LVH owing to compressive loading and augmented systolic wall stress. Furthermore, our study revealed significant LA remodeling in the REG, potentially ascribed to elevated LVMi and LVEDV [41]. Although the mechanisms underpinning atrial adaptation to exercise remain incompletely understood [42], emerging evidence suggests that LA may be more responsive to exercise than LV [43]. The adaptive capacity of LA serves to modulate LV filling pressures, both at rest and during physical exertion [44].

Notably, our analysis revealed no significant differences between the groups regarding aortic stiffness. Established cardiovascular risk factors, including age, diabetes, hypertension, smoking, and dyslipidemia, exacerbate arterial stiffness and promote atherosclerosis [45]. Nevertheless, regular engagement in either aerobic or resistance exercise appears to exert comparable salutary effects on cardiovascular stiffness and elasticity [46, 47].

Despite the absence of significant differences in diastolic echocardiographic parameters between the groups, IVRT was within normal limits but protracted in the REG, possibly due to the heightened LVMi [48]. This may also explain the observed elongation of IVRT in the absence of pathological diastolic dysfunction [48]. In addition, the higher LATEVi, LAmaxi, and LAPi parameters suggest that RE may have a significant effect on LA. However, during RE, blood pressure and pulse fluctuations may occur because of vagal activity and increased intrathoracic pressure. Although no disturbance was noted in the diastolic parameters, further investigations should be conducted to determine whether this is a promoting factor, particularly for arrhythmias of atrial origin.

In summary, our findings emphasize the distinctive cardiac adaptations associated with different exercise modalities, with resistance training in particular inducing notable alterations in LV and LA parameters [49, 50]. While these adaptations are generally physiological and beneficial, ongoing surveillance is prudent, particularly to discern any potential pro-arrhythmic effects, especially of atrial origin, under certain conditions [51]. Further research is warranted to substantiate these observations and elucidate the long-term clinical implications of exercise-induced cardiac adaptations.

This study has certain limitations that should be considered when interpreting the findings. First, our investigation focused on male individuals who engaged in recreational sports and those who performed RE. Therefore, the generalizability of the results to other populations or individuals participating in different types of exercises may be limited. Future studies should explore the impact of various exercise modalities on cardiac parameters to obtain a more comprehensive understanding. Second, it is important to acknowledge that our study design was cross-sectional, which does not explain the causation underlying adaptation. Longitudinal cohort studies with extended follow-up periods are necessary to assess the long-term effects of exercise on the cardiac structure and function. Such studies would allow the investigation of potential structural changes in the heart over time and provide valuable insights into the progression of cardiovascular adaptations associated with exercise. Third, this study focused on the effects of specific training but did not consider individual differences in training intensity, duration, frequency, and baseline level of cardiorespiratory fitness among the participants. This variability in training could potentially have influenced the outcomes of this study. These limitations highlight the need for further research that includes a more comprehensive evaluation of training variables and baseline fitness levels, which can provide a greater understanding of the mechanisms of cardiac modulation and other relevant outcomes. Despite these limitations, our study contributes to the existing body of knowledge regarding the effectiveness of RE and recreational football training in cardiac adaptation.

## Conclusion

According to the findings of our study, we observed notable variations in the LV and LA functions in the REG compared to the RSG. These disparities were evident despite the weekly exercise frequency and volume being comparable between the cohorts. Our data suggest that resistance exercises (REs) induce significant cardiac remodeling, which is characterized by alterations in myocardial structure and function. While recreational exercises are documented to enhance overall physical fitness levels [7], our findings indicate that their impact on cardiac adaptation is not as pronounced as that observed with resistance exercises. Consequently, we propose that individuals engaging in exercise for recreational purposes should consider incorporating exercise modalities that impose greater cardiovascular demand, thereby promoting more substantial cardiac conditioning. Furthermore, we advocate the involvement of an exercise physiologist or a clinical exercise specialist in the design and supervision of exercise regimens for these individuals to ensure safety and maximal therapeutic benefit.

## Data Availability

The data is not publicly available because further research is being done and more manuscripts are being prepared. Data for the current study will be available upon reasonable request from the principal investigator or corresponding author.

## Abbreviations

A: Late diastolic flow parameter
Am: Peak late diastolic
AD: Aortic distensibility
ADd: Aortic diameter diastole
ADs: Aortic diameter systole
AS: Aortic strain
BMI: Body mass index
BSA: Body surface area
CVi: Conduit volume index
DBP: Diastolic blood pressure
E: Early diastolic flow velocity
Em: Peak early diastolic
ECHO: Echocardiography
ET: Ejection time
IVCT: Isovolumic contraction time
IVRT: Isovolumic relaxation time
IVST: Interventricular septal thickness
LVEF: Left ventricular ejection fraction
LVMi: Left ventricular mass index
LAAEF: Left atrium active emptying fraction index
LAAEV: Left atrium active emptying volume index
LAPEF: Left atrium passive emptying fraction index
LAPEV-I: Left atrium passive emptying volume index
LATEF-I: Left atrium total emptying fraction index
LATEFSV-I: Left atrium total emptying fraction index
LVDd: Left ventricular end-diastolic diameter
LVDs: Left ventricular end-systolic diameter
LVEDVi: Left ventricular end-diastolic volume index
LVESVi: Left ventricular end-systolic volume index
PWT: Posterior wall thickness
REG: Resistance exercise group
RSG: Recreational sports group
Sm: Peak early systolic
SVi: Stroke volume index
SBP: Systolic blood pressure
TF: Training frequency
